# Ribose-cysteine and levodopa abrogate Parkinsonism via the regulation of neurochemical and redox activities in alpha-synuclein transgenic *Drosophila melanogaster* models

**DOI:** 10.1080/19336934.2024.2306687

**Published:** 2024-01-29

**Authors:** Olumayowa K. Idowu, Ademola A. Oremosu, Olufunke O. Dosumu, Abdullahi A. Mohammed

**Affiliations:** a Department of Anatomy, College of Medicine, University of Lagos, Lagos, Nigeria; b Department of Anatomy, College of Health Sciences, Osun State University, Osogbo, Nigeria; c Department of Human Anatomy, School of Medicine and Pharmacy, College of Medicine and Health Sciences, University of Rwanda, Rwanda

**Keywords:** Alpha-synuclein, *Drosophila melanogaster*, levodopa, neurochemical, Parkinsonism, ribose-cysteine, transgenic

## Abstract

Parkinson’s disease (PD), the most prevalent type of parkinsonism, is a progressive neurodegenerative condition marked by several non-motor and motor symptoms. PD is thought to have a complex aetiology that includes a combination of age, genetic predisposition, and environmental factors. Increased expression of α-synuclein (α-Syn) protein is central to the evolvement of neuropathology in this devastating disorder, but the potential of ribose-cysteine and levodopa in abating pathophysiologic changes in PD model is unknown. Crosses were set up between flies conditionally expressing a pathological variant of human α-Syn (UAS-α-Syn) and those expressing GAL4 in neurons (elav-GAL4) to generate offspring referred to as PD flies. Flies were randomly assigned to five groups (*n* = 40) from the total population of flies, with each group having five replicates. Groups of PD flies were treated with either 500 mg/kg ribose-cysteine diet, 250 mg/kg levodopa diet, or a combination of the two compounds for 21 days, whereas the control group (w^1118^) and the PD group were exposed to a diet without ribose-cysteine or levodopa. In addition to various biochemical and neurochemical assays, longevity, larval motility, and gravitaxis assays were carried out. Locomotive capability, lifespan, fecundity, antioxidant state, and neurotransmitter systems were all significantly (*p* < 0.05) compromised by overexpression of α-Syn. However, flies treated both ribose cysteine and levodopa showed an overall marked improvement in motor functions, lifespan, fecundity, antioxidant status, and neurotransmitter system functions. In conclusion, ribose-cysteine and levodopa, both singly and in combination, potentiated a therapeutic effect on alpha-synuclein transgenic *Drosophila melanogaster* models of Parkinsonism.

## Introduction

Parkinsonism is a term that collectively describes many brain disorders that result in slowed movements, rigidity, and tremors associated with Parkinson’s disease (PD). Many factors including genetic modifications, adverse drug reactions, and infections are associated with the cause of these disorders [1,2]. PD, which is the most common form of parkinsonism [3], is a progressive neurodegenerative disorder characterized by different forms of non-motor and motor symptoms [4], occurring due to the dysfunction of various neurotransmitter pathways, including the dopaminergic, serotonergic, and cholinergic systems, among others [5]. A major trait of the neuropathology of PD is the misfolding and intracellular accumulation of α-synuclein (α-Syn) [6].

The interplay of ageing, genetic predisposition, and environmental factors is likely to be one of the many factors contributing to the aetiology of PD [7]. However, the α-Syn protein is essential for the pathophysiology of the disease. PD leads to motor deficits such as bradykinesia, stiffness, and tremors at rest, although non-motor symptoms such as gastrointestinal disorder, loss of smell, sleep problems, and reproductive health issues may appear during a protracted prodromal period before the onset of the motor features [3].

The synuclein alpha (SNCA) gene, which encodes for α-Syn, was the first gene shown to be connected to the molecular aetiology of PD. The SNCA gene mutation is a genetic causative factor of PD [8,9]. Although *Drosophila melanogaster* (*D. melanogaster*) has no known homolog of α-Syn, key hallmarks of PD, including Lewy body-like aggregation and abnormal locomotor behaviour, have been replicated in flies by overexpressing transgenes of wild-type and mutant α-Syn [10,11]. Due to its high rate of reproduction, brief life cycle, and few maintenance requirements, *D. melanogaster* has been used in behavioural and medical research for many years. It also has orthologs for around 75% of human disease genes [12,13]. Moreover, due to its adherence to the 3Rs requirements, it has received approval from the European Center for the Validation of Alternative Models, and it has fewer ethical issues than rats and other higher animals [12,14].

Levodopa, a pro-dopamine medication produced via biosynthesis from L-tyrosine, is used to raise dopamine levels in the treatment of PD [15], although long-term dopamine replacement therapy insensitivity is still a drawback. Mitigation of PD and reversal of neurodegeneration are possible if there is a mechanism to savage free radicals and restore synaptic connections between neurons. Therefore, there is a need for a product that can be used to manage the disease over a long period of time without side effects. Hence, ribose-cysteine, a glutathione booster, was explored in this study.

Ribose-cysteine is a prodrug of cysteine designed to increase the intracellular biosynthesis of glutathione in live cells. The tri-peptide antioxidant glutathione is a substance that physiologically supports cellular homoeostasis [16]. The rate-limiting factor in the synthesis of glutathione, which is composed of glutamic acid, cysteine, and glycine, is the amino acid cysteine. Consequently, the availability of cysteine affects how much glutathione is present in tissues [17]. Studies in the past have demonstrated that oral glutathione delivery is ineffective because it is considerably broken down by digestive enzymes [18]. In light of this, the ribose sugar component of the ribose-cysteine used in the current study aids in the protection and biological transport of cysteine to the cells, thereby increasing the biosynthesis, release, and physiological usage of glutathione [19].

In the current study, we have used the GAL4/UAS system to determine the potential beneficial effects of ribose-cysteine and levodopa, singly and in combination, on parkinsonism-like outcomes in α-Syn transgenic *D. melanogaster* via the determination of lifespan, locomotor performance, offspring emergence rate, oxidant/antioxidant status, and neurotransmitter levels.

## Materials and methods

### Chemicals

This study employed analytical-grade chemicals and reagents throughout. Ribose-cysteine (99% purity) and levodopa (99% purity) were procured from Max International Oral Company in the United States and Shaanxi Phoenix Tree Biotech Co., Ltd., respectively. Other chemicals and reagents were sourced locally.

### Drosophila melanogaster stock and *culture*

The genetically altered *D. melanogaster* w^1118^ (#3605), w[*]; P{w[+mC] = UAS-Hsap\SNCA.F}5B (#8146), and w[*]; P{w[+mC] = GAL4-elavL}3 (#8760) were gifted by Dr. I. O. Ishola from the Pharmacology and Therapeutics Department, University of Lagos, Nigeria. All the flies originated from the Bloomington *Drosophila* Stock Centre (Indiana University, Bloomington, IN). The flies were kept and raised in the Drosophila Laboratory, Anatomy Department, University of Lagos, Nigeria, on cornmeal feed mixed with brewer yeast (1% w/v), agar (1% w/v), and nipagin (0.8% v/w), under standard conditions of a 12-hour cycle of darkness and light with consistent temperature and humidity (22–24°C and 60–70%, respectively) [20].

### Drosophila melanogaster strain *crosses*

To express α-Syn in elav-GAL4, virgin w[*]; P{w[+mC] = GAL4-elavL}3 (elav-GAL4) females were collected within one to three hours of their emergence from the vials; they were then exposed to males of the w[*]; P{w[+mC] = UAS-Hsap\SNCA.F}5B (UAS-α-Syn) strains at a ratio of one to three (male/female; 1:3). These crosses gave rise to offspring referred to as PD flies because they had neurons in which α-Syn transgenes were expressed. Moreover, it has been reported in previous studies that similar crosses gave rise to offspring expressing locomotor dysfunctions, oxidative stress, poor fecundity, and reduced longevity [14,21,22].

### Determination of the effective dose of ribose-cysteine and levodopa

The most appropriate dose of ribose-cysteine was established following the approach of Olanrewaju et al. (2023), with slight changes. PD flies that were one to three days old were separated into four groups and given 14 days of exposure to a diet mixed with ribose-cysteine at doses of 0, 125, 250, or 500 mg/kg. Daily mortality was monitored, and for the survivors, a gravitaxis test was performed. The 500 mg/kg ribose-cysteine diet was shown to be the most efficient dose at the conclusion of this phase of the experiment [23]. Outcomes of our pilot studies revealed that concentrations of ribose-cysteine lower than 125 mg/kg diet had no significant physiological relevance, while concentrations above 500 mg/kg diet did not bring any better outcomes. The adopted dose of levodopa was the effective dose previously determined [24].

### Experimental design

Flies that were between one and three days old were placed into five groups (Control; PD; PD + RC; PD + LD; and PD + RC + LD), each containing 40 flies and having five replicates. PD flies were exposed to a 500 mg/kg ribose-cysteine diet (PD + RC), 250 mg/kg levodopa diet (PD + LD) or a combination of 500 mg/kg ribose-cysteine diet and 250 mg/kg levodopa diet (PD + RC + LD). The control group (w^1118^) and the untreated PD group (PD) were exposed to a diet without ribose-cysteine or levodopa. Every other day, a freshly prepared diet was given to the flies, throughout the entire duration of treatment. On day 21, following lifespan and gravitaxis assays, the flies were anesthetized with a fly nap, weighted, homogenized at a 1 mg:10 μL ratio in 0.1 M phosphate-buffered saline (pH 7.2), and then centrifuged at 4°C for 15 minutes [20]. The obtained sample supernatants were placed into Eppendorf tubes, where they were utilized to assess neurochemical parameters as described. A subset of treated flies was used for larval motility assay, and the rate at which the offspring emerged from the vials was recorded.

### Larval locomotor function assays

The larval mobility test was conducted using roving third-instar larvae as previously described [25]. A grid with 1 cm^2^ boxes was set on top of a Petri dish containing 3% agarose, and the number of lines that the head of the larvae crossed in 30 seconds was recorded. Larvae were allowed to undergo three trials, and the data from the final experiment was used in the study.

### Gravitaxis test for locomotor function

For the Gravitaxis test, twenty flies were placed in vertical tubes in each group and given 15 minutes to acclimate. The tube was then turned upside down, and the flies were gently tapped to the base. Within 8 seconds, the number of flies that climbed the tube to a height of 17.5 cm was counted. The performance index (PI) was determined by calculating the percentage of all flies that climbed to the top of the tube. This technique was performed three times at one-minute intervals, and the data were recorded for analysis [26].

### Eclosion assay

At the end of the experiment, the vials in which the flies were raised were collected and monitored daily for larval development, pupation, and eclosion. The rate of emergence was calculated based on the number of adult flies that successfully emerged from the pupal case. Every fly that emerged from each vial was counted for 21 days and recorded for analysis [27].

### Survival assay

Longevity was assessed using the method previously reported by Farombi et al. (2018). A score of 1 was given for each daily death event, whereas a score of 0 was given for no-death events. Up until the last fly was dead, records were kept throughout the experiment, and the data were plotted on a survival curve [27].

### Determination of dopamine, serotonin and acetylcholine concentrations

Following the procedure described by the manufacturer, the concentrations of dopamine, serotonin, and acetylcholine in pulverized fly samples were estimated using the ELISA kit (Elabscience, China). Using a Spectramax Plus 384 microplate reader, the absorbance was calculated at 450 nm.

### Determination of malondialdehyde (MDA) level

The supernatant from the fly homogenate was spectrophotometrically examined to assess MDA levels using an adjusted form of the procedure outlined by Karatas et al. (2002). Fly eyes were previously removed to prevent eye pigment from affecting MDA absorbance. A petri dish filled with phosphate-buffered saline was used to hold the anesthetized flies under the dissecting microscope, and the reddish coverings on the eyes were peeled off using the dissecting tweezers [28]. The results were given as µmol/ml.

### Measurement of nitrite and nitrate as indices of nitric oxide (NO) level

The level of NO was determined using Griess reagent. The theory is based on the enzymatic conversion of nitrate to nitrite. The Griess reagent was incubated with the samples in a 1:1 ratio for 20 minutes at 25°C, and the absorbance was measured at 595 nm. The concentration of NO in each sample was obtained using a NaNO_3_ standard curve [11,29].

### Determination of glutathione S-transferase (GST) activity

The method described by Habig and Jakoby (1981) was used to measure the enzymatic activity of GST. 270 μL of a solution containing 20 μL of 0.25 M potassium phosphate buffer, pH 7.0, 2.5 mM EDTA, and 10.5 μL of distilled water, together with 10 μL of 25 mM 1-chloro-2,4-dinitrobenzene (CDNB), 20 μL of sample (1:5), and 25 μL of 0.1 M GSH at 25°C, made up the reaction mixture. Spectrophotometry was used to read the final solution for 5 minutes (10 s at a time) at 340 nm [30].

### Determination of acetylcholine esterase (AChE) activity

AChE activity was assessed following the procedure outlined by Ellman et al. (1961). A buffer containing 0.1 M potassium phosphate, pH 7.4, 1 mM DTNB, and 0.8 mM acetylthiocholine as the initiator was used in this experiment. At 30-second intervals for two minutes, the response was observed at 412 nm [31].

### Determination of total thiol content

Following the approach stated by Ellman (1959), total thiol content was measured. The reaction mixture included 20 μL of homogenate supernatant, 35 μL of 1 mM DTNB, and 35 μL of distilled H_2_O in 510 μL of 0.1 M phosphate buffer (pH 7.4). The incubation process was then carried out for 30 minutes at 25°C, and the absorbance was measured at 412 nm [32].

### Determination of cell mitochondrial metabolic rate (cell viability)

Cell viability was evaluated by utilizing the 3-(4,5-dimethylthiazol-2-yl)-2,5-diphenyltetrazolium bromide (MTT reduction test). The samples were incubated in MTT (500 μg/ml) for 60 min (37°C) and then incubated in DMSO for 30 minutes at 37°C after washing off excess MTT. A spectrophotometer plate reader was used to measure the coloured formazan salt at 590 nm [33,34].

### Statistical analysis

The obtained data were expressed as the mean ± SEM. Graph Pad Prism 8 was used for statistical analysis and to created graphs (Graph Pad Software Inc., USA). Depending on the variable tested, one- or two-way ANOVA was used with Tukey’s multiple comparisons test. *p* < 0.05 was used to determine significance.

## Results

### Determination of effective dose of ribose-cysteine in *D. melanogaster* parkinsonism model

**Figure 1.**
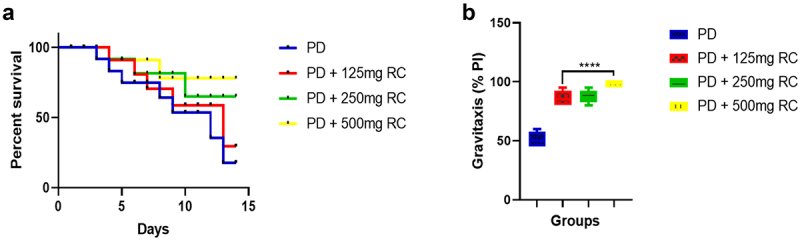
Percentage survival at varied ribose-cysteine concentrations (a): ribose-cysteine prolonged longevity in a dose-dependent manner. Gravitaxis performance index on varied ribose-cysteine exposure (b): there is a significant increase in gravitaxis performance in all the PD groups exposed to ribose-cysteine compared to the group without ribose-cysteine treatment. Data is presented as mean ± S.E.M. at ****: *p* < 0.0001 compared to PD. (RC: ribose-cysteine, PD: PD flies).

Exposure of flies to 125, 250, and 500 mg/kg ribose-cysteine diets for 14 days increased longevity by 29.50%, 65.20%, and 77.90%, respectively, and increased gravitational performance compared to the PD flies that did not receive ribose-cysteine (Figures 1(a,b)). 500 mg/kg ribose-cysteine diet was considered for the main experiment given that the highest survival rate and highest gravitaxis performance were recorded at this concentration.

### Ribose-cysteine and levodopa improves locomotor functions, rate of offspring emergence, and survival in Parkinsonian *D. melanogaster*

**Figure 2.**
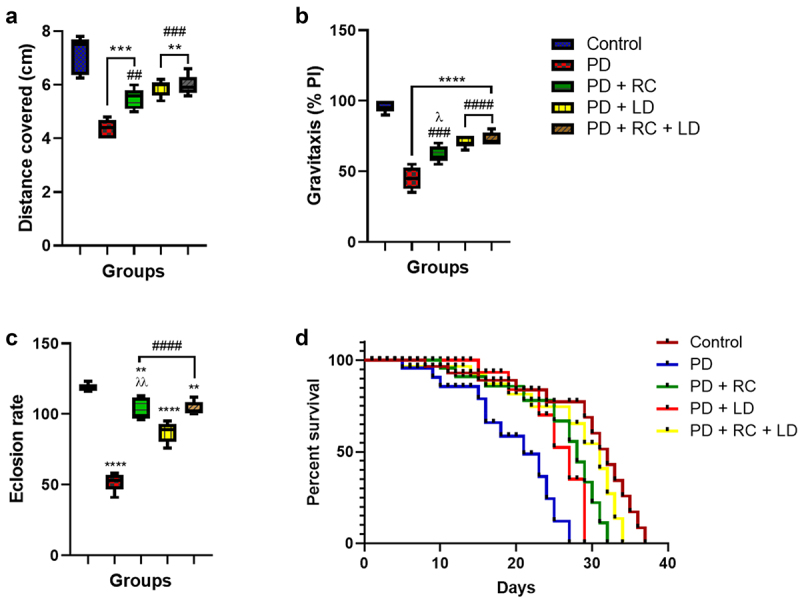
Distance covered on the larval motility assay (a), performance index on the gravitaxis assay (b), number of offspring emergence (c), and survival assay (d). There was a statistically significant decrease in larval motility, adult gravitaxis, and eclosion rate in all the PD groups compared to the control. On the other hand, all the PD groups exposed to ribose-cysteine and levodopa showed a statistically significant increase in these parameters when compared to the PD group fed on a normal diet. On the survival assay, the highest longevity was observed in the control group, while the groups exposed to ribose-cysteine and levodopa showed extended longevity over the PD group which were on a normal diet. The result is expressed as mean ± S.E.M. at **: *p* < 0.01, ****: *p* < 0.0001 compared to control; ##: *p* < 0.01, ###: *p* < 0.001, ####: *p* < 0.0001 compared to PD; λ: *p* < 0.05, λλ: *p* < 0.01 compared to PD + RC + LD (control: w^1118^, RC: ribose-cysteine, LD: levodopa, PD: Parkinson’s disease).

A significant reduction in larval motility (*p* < 0.01, *p* < 0.0001) and gravitaxis performance (*p* < 0.0001) was observed in the entire PD fly group compared to the control. Conversely, a significant improvement in larval motility and adult gravitaxis was observed in Parkinsonian flies treated with ribose-cysteine (*p* < 0.01, *p* < 0.001) and levodopa (*p* < 0.001, *p* < 0.0001) compared to the PD group fed on a normal diet. Moreover, the PD + RC group showed markedly (*p* < 0.05) reduced gravitaxis performance compared to the PD + RC + LD group (Figures 2(a,b)). Further, we found a significant reduction in the rate of offspring emergence in the PD flies compared to the control (*p* < 0.01, *p* < 0.0001; Figure 1(d)). However, all the ribose-cysteine and levodopa-treated Parkinsonian flies showed markedly increased rate of offspring emergence compared to the PD group fed on normal diet (*p* < 0.0001). Furthermore, a significant (*p* < 0.05) decrease in the rate of offspring emergence was observed in the PD + LD group compared to the PD + RC + LD group (Figure 2(c)). There was also a 29.7%, 10.8%, 13.5%, and 2.7% reduction in survival rate in the PD, PD + RC, PD + LD, and PD + RC + LD groups, respectively, compared to the control. However, the PD + RC, PD + LD, and PD + RC + LD groups showed about 18.9%, 16.5%, and 27.3% increases in survival rate, respectively, when compared to the PD group fed on a diet without ribose-cysteine/levodopa (Figure 2(d)).

### Ribose-cysteine and levodopa regulates neurotransmitters (dopamine, serotonin and acetylcholine) activities in parkinsonian *D. melanogaster*

**Figure 3.**
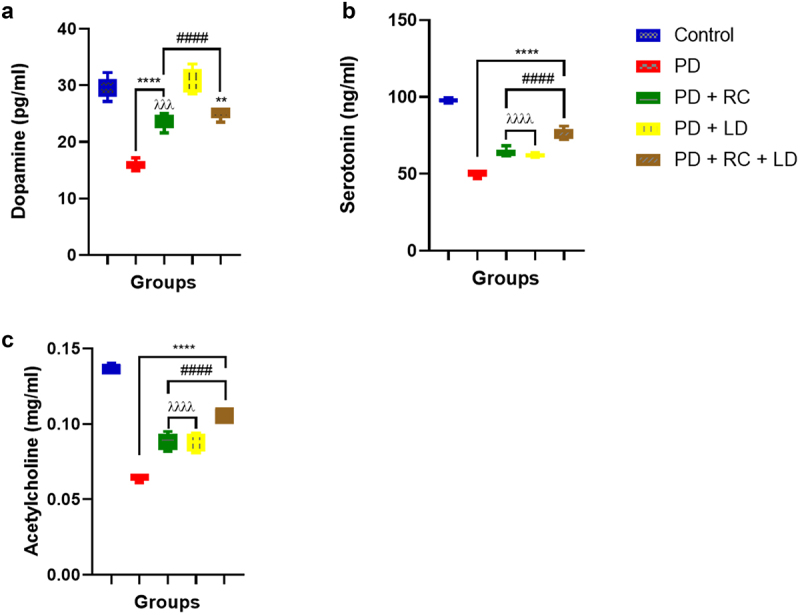
Dopamine level (a), serotonin level (b), and acetylcholine level (c). Compared to control, the levels of dopamine in the PD, PD + RC, and PD + RC + LD groups decreased significantly, but not in the PD + LD group. Further, a statistically significant increase in the levels of dopamine was observed in all the groups exposed to ribose-cysteine and levodopa compared to the PD group fed on a normal diet. Moreover, serotonin and acetylcholine levels were significantly lower in all the PD groups compared to the control. A significant increase in the levels of serotonin and acetylcholine was observed in all the groups exposed to ribose-cysteine and levodopa compared to the PD group which were on normal diet. The analysis is presented as mean ± S.E.M. at **: *p* < 0.01, ****: *p* < 0.0001 compared to control; ####: *p* < 0.0001 compared to PD; λλλ: *p* < 0.001, λλλλ: *p*< 0.0001 compared to PD + RC + LD (control: w^1118^, RC: ribose-cysteine, LD: levodopa, PD: PD flies).

It was observed in this result that the flies in the PD (*p* < 0.0001), PD + RC (*p* < 0.0001), and PD + RC + LD (*p* < 0.01) groups showed significantly reduced levels of dopamine compared to the control group, whereas no significant difference was observed between the PD + LD group and the control. Conversely, flies in all the ribose-cysteine/levodopa-treated groups showed elevated (p < 0.0001) levels of dopamine compared to the PD group that was fed a normal diet. Moreover, the ribose-cysteine-treated PD group demonstrated a significantly decreased level of dopamine compared to the ribose-cysteine and levodopa-co-treated PD group (p < 0.001; Figure 3(a)). Also, the levels of serotonin and acetylcholine were concurrently reduced in all the PD groups compared to the control (p < 0.0001). Conversely, flies in all the ribose-cysteine/levodopa-treated groups showed elevated levels of serotonin and acetylcholine compared to the PD group fed a normal diet (p < 0.0001). Furthermore, the PD + RC and PD + LD groups showed significantly (p < 0.0001) lowered levels of serotonin and acetylcholine compared to the PD + RC + LD group (Figure 3(b,c)).

### Ribose-cysteine and levodopa improved redox parameters in Parkinsonian *D. melanogaster*

**Figure 4.**
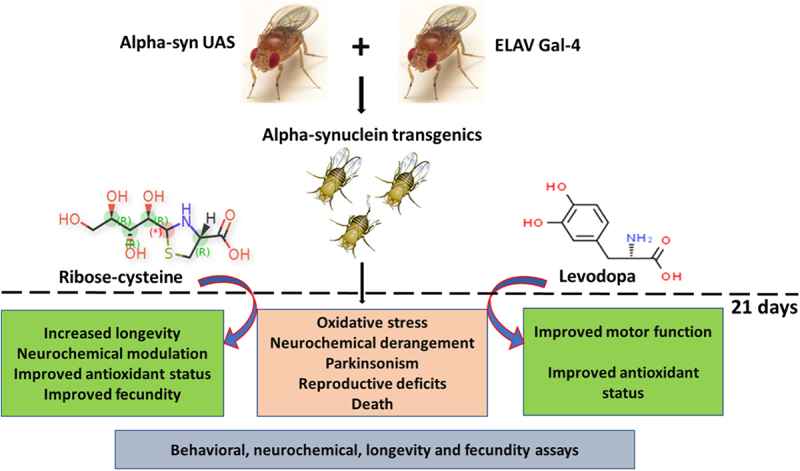
Schematic illustration of the research protocol to study ribose-cysteine and levodopa effect on alpha-synuclein transgenic *Drosophila melanogaster*.

**Figure 5.**
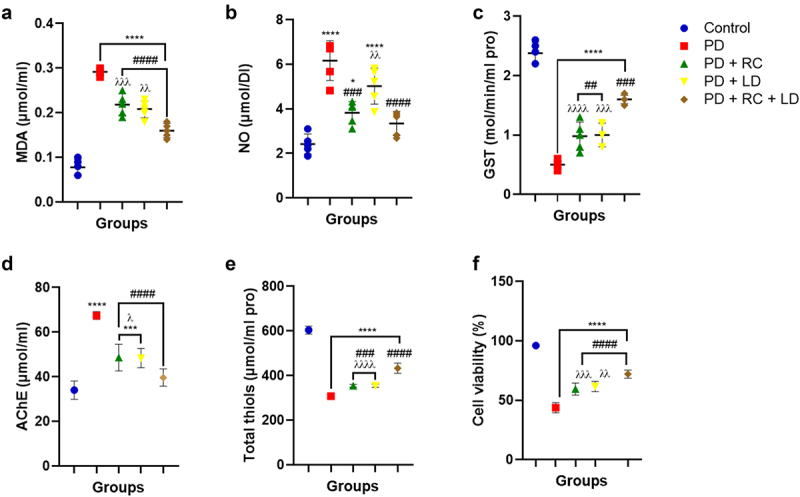
Malondialdehyde level (a), nitric oxide level (b), glutathione S-transferase activity (c), acetylcholine esterase activity (d), total thiol level (e), and cell viability (f). A significant increase in the level of MDA was observed in all the PD groups, as well as a significantly increased level of NO in the PD, PD + RC, and PD + LD groups, but not in the PD + RC + LD group compared to the control. However, the level of MDA was significantly decreased in all the groups exposed to ribose-cysteine and levodopa compared to the PD group fed on a diet without ribose-cysteine or levodopa, as well as decreased NO levels in the PD + RC and PD + RC + LD groups, but not in the PD + LD group compared to the PD group fed on a diet without ribose-cysteine or levodopa. Moreover, there is a statistically significant decrease in GST activity in all the PD groups, as well as a significantly elevated AChE activity in the PD, PD + RC, and PD + LD groups, but not in the PD + RC + LD group compared to the control. However, GST activity was significantly increased, with decreased AChE activity in all the groups exposed to ribose-cysteine and levodopa compared to the PD group fed on a diet without ribose-cysteine or levodopa. Also, there is a statistically significant concurrent decrease in total thiol level and percentage cell viability observed in all the PD groups compared to the control. Conversely, there is a statistically significant increase in total thiol level and percentage cell viability in all the groups exposed to ribose-cysteine and levodopa compared to the PD group fed on a diet without ribose-cysteine or levodopa. The result is expressed as mean ± S.E.M. at **: *p* < 0.01, ****: *p* < 0.0001 compared to control; ####: *p* < 0.0001 compared to PD; λλλ: *p* < 0.001, λλλλ: *p* < 0.0001 compared to PD + RC + LD. (control: w^1118^, RC: ribose-cysteine, LD: levodopa, PD: PD flies).

The results showed that the MDA levels in all the PD groups increased statistically significantly (*p* < 0.0001), and the NO levels in the PD, PD + RC, and PD + LD groups elevated statistically significantly (*p* < 0.05, *p* < 0.0001) when compared to the control. However, there was no significant difference observed between the PD + RC + LD group and the control. Conversely, when compared to the PD group fed on a diet without ribose-cysteine/levodopa, flies in all ribose-cysteine/levodopa-treated groups showed significantly (*p* < 0.0001) decreased MDA as well as decreased (*p* < 0.001, *p* < 0.0001) NO levels in PD + RC and PD + RC + LD. There was no statistically significant difference between the PD + LD and PD groups given a diet devoid of levodopa or ribose-cysteine. But as compared to the PD + RC + LD group, the PD + LD and PD + RC groups showed a considerable (*p* < 0.01, *p* < 0.001) elevation in MDA, and the PD + LD group also had a considerably (*p* < 0.01) elevated NO level. Furthermore, the NO assay did not reveal a statistically significant difference between the PD + RC + LD and control groups, nor between the PD + LD group and the PD group fed a diet devoid of ribose-cysteine/levodopa (Figure 4(c,d)). All of the PD groups showed a statistically significant (*p* < 0.0001) decrease in GST activity, while the PD, PD + RC, and PD + LD groups showed significantly (*p* < 0.001, *p* < 0.0001) increased AChE activity compared to the control. In contrast, flies in all ribose-cysteine/levodopa-treated groups showed a significant increase in GST activity (*p* < 0.01, *p* < 0.001) and a significant decrease in AChE activity (*p* < 0.0001) when compared to the PD group that was fed a diet free of ribose-cysteine/levodopa. When compared to the PD + RC + LD group, the PD + LD and PD + RC groups showed significantly (*p* < 0.05) elevated AChE activity and significantly (*p* < 0.01) reduced GST activity. Regarding AChE activity, there was no statistically significant difference between the control and PD + RC + LD groups (Figure 5(a,b)).

### Ribose-cysteine and levodopa improved total thiols and mitochondrial metabolic activity in Parkinsonian *D. melanogaster*

When compared to the control, we found a significant concurrent decrease in total thiol level and percentage cell viability in all the PD groups (p < 0.0001). On the other hand, flies in all the ribose-cysteine/levodopa-treated groups showed significantly elevated total thiol levels (p < 0.0001) and percentage cell viability (p < 0.001, p < 0.0001) compared to the PD group fed normal diet. The PD + RC (p < 0.001) and PD + LD (p < 0.0001) groups further showed significantly lowered levels of total thiols, and percentage cell viability (both at p < 0.0001) compared to the PD + RC + LD group (Figure 5).

## Discussion

The pathogenic characteristic that distinguishes PD and other synucleinopathies is α-Syn accumulation and aggregation [35]. Prior to the death of neurons, synaptic impairments and consequent synaptopathy are correlated with α-Syn aggregation [36]. The processes and sequence of events that underlie the pathogenic transition from α-Syn build-up to synaptopathy and ensuing neurodegeneration, however, are not fully known [35]. In the current study, *D. melanogaster*, a well-known model for studying PD and synucleinopathy, was used to assess the effect of ribose-cysteine co-treatment with levodopa on α-Syn-mediated parkinsonism.

The concentrations of ribose-cysteine that elicits therapeutic effects range between 125 and 500 mg/kg diet, and this study provides a novel indication that 500 mg/kg ribose-cysteine in diet may be the optimal dose of ribose-cysteine with therapeutic impact in a in *D. melanogaster* Parkinsonism model.

Based on the findings of this study, α-Syn greatly reduced the larval and adult locomotive capacity and lifespan of the PD flies. These phenotypic expressions are in agreement with the reports of Vicente-Miranda et al. (2017), Siddique et al. (2021), and Ishola et al. (2022), who reported similar outcomes in these transgenic strains [22,37,38]. Interestingly, the present study is contrary to the report by Mohite et al. (2018) which showed that locomotor and life span changes are not observed in these strains [39]. While the reasons for the discrepancies in the phenotypes expressed by the PD flies in different literatures are not clearly identified, there are several factors, including diet, geographical location, climatic conditions, and individual idiosyncratic behaviours, that may affect experimental outcomes [40,41]. The build-up of aggregated α-Syn in the mitochondria of dopaminergic neurons and α-Syn-associated ferroptosis, which is implicated in the susceptibility of the neurons to oxidative stress-induced death [42–44] are attributable to the emergence of motor dysfunction observed in this study. This is consistent with the observation that motor neuron dysfunction and degeneration are linked to the aggregation of α-Syn [45]. When overexpressed, α-Syn localizes exclusively to the nerve terminal, limits neurotransmitter release, and generates cytotoxicity, thus disrupting the normal activity of the neurons and ultimately resulting in neuronal damage and degeneration [46,47]. The shorter lifespan of the PD flies in this study may be due to disruption of the physiological activity of α-Syn in neurotransmission [48], leading to impairment of mitochondrial structure and functions and oxidative stress-induced death [43,49]. Previous findings have suggested a connection between thalamic dopamine deficiency in PD and impaired activation of the hormones responsible for reproduction, such as oestrogen and testosterone [50,51]. Although it is not well elucidated in literature, it could be suggested that fly reproductive hormones such as ecdysone and juvenile hormones [52] may be impaired by dopamine deficiency.

However, ribose-cysteine, levodopa, and a combination of the two offered a defence against the α-Syn-induced locomotor deficits and reduced fecundity and survival rate in the treated flies. The enhanced locomotor performance, improved fecundity, and extended survival in response to ribose-cysteine treatment underlie its role in the antioxidant defence system to prevent oxidative stress [53]. In agreement with the results of this study, levodopa has been reported in previous studies to improve locomotor activity, lifespan [24], and reproductive performance [54].

In *D. melanogaster*, neurotransmitters such as dopamine, serotonin, and acetylcholine are involved in a wide variety of behaviours [55]. The release of these neurotransmitters may be disrupted by aggregated forms of α-Syn, causing neurotoxicity [46,56]. The concurrently low levels of the neurotransmitters in the PD flies are traceable to the inhibitory effect of α-Syn on synaptic vesicle re-clustering following endocytosis [57], leading to motor and non-motor deficits [58]. Additionally, significant loss of brain dorsomedial dopaminergic neurons has been reported in α-Syn transgenic PD flies [14,59], buttressing the low level of dopamine observed in this study. While an increased acetylcholine level was expected to accompany dopamine deficiency [60], this study demonstrated a concomitant dopamine and acetylcholine deficiency. Although this discrepancy is not clearly understood, low acetylcholine is reportedly associated with non-motor symptoms of PD [61]. Moreover, the advanced stage of PD is associated with concurrent loss of dopamine and acetylcholine [62]. Interestingly, ribose-cysteine and levodopa monotherapy and combined therapy protected against the α-Syn-induced disruption of neurotransmitter activities. While the specific process involved is not well elucidated, glutathione undoubtedly plays an important role in the brain as a neuromodulator [63]. Hence, the ability of ribose-cysteine to modulate the neurotransmitters dopamine, serotonin, and acetylcholine further substantiates its glutathione-boosting property. Through its reducing actions, glutathione is known to restore synaptic plasticity mechanisms by regulating the activation of N-methyl-D-aspartate receptors, which are crucial to synaptic plasticity [64]. Levodopa is a known dopamine precursor; it increases dopamine neurotransmission by crossing the blood-brain barrier and increasing dopamine concentrations [65]. According to the literature, levodopa is expected to increase dopamine concentration, thereby reducing serotonin levels in the brain [66,67]. However, the elevated level of serotonin on levodopa treatment in the present study is still largely unclear and calls for further investigation.

It is a fact that overexpression of the α-Syn gene leads to overproduction of free radicals in genetic models of neurodegenerative diseases [68]. Accumulation of MDA, a secondary product of lipid peroxidation, is a clear indicator of cellular injury [11,69], while overproduction of NO is associated with the trigger of pro-inflammatory activation [29], and elevated activity of AChE has been linked to the pathophysiology of PD [11,70]. According to the results of the current study, the levels of MDA, NO, and AChE activity were raised concurrently in the PD flies as a result of α-Syn aggregation. The simultaneously elevated levels of MDA and NO found here are traceable to the breakdown of the tissue’s lipid components and DNA integrity, as well as the start of various inflammatory processes associated with α-Syn accumulation [71], while the increased AChE activity is most probably consequential to low acetylcholine levels.

One of the many tissue innate protections against oxidative damage is GST, a significant detoxifying enzyme that is mostly located in the cytosol [72] and catalyses the synthesis of glutathione [73]. Low-molecular-weight and protein-based redox systems both heavily depend on thiols. Thiol-based antioxidants are essential for detoxifying and preserving the antioxidant status of the cell [74,75]. Cell viability is a measure of the structural and functional integrity of the cell and its ability to respond to outside stimuli, chemicals, or medical treatments [76]. As observed in this study, reduced cell viability, low thiol levels, and suppression of GST activity are all associated with PD [20], and the functions of ribose-cysteine and levodopa in reducing these neurotoxic changes is an important underpinning of the therapeutic potentials of both compounds in the *D. melanogaster* Parkinsonism model.

An important mechanisms of α-Syn-induced neurotoxicity in the PD flies is via the induction of oxidative stress, which is linked to excessive free radical production [68]. Our current reports found that ribose-cysteine and levodopa however decreased the elevated MDA and NO levels in PD flies, alongside a marked decrease in AChE activity. We further found that ribose-cysteine improves GST activity and increases total thiol levels and cell viability in the PD model. These effects could be traceable to ribose-cysteine’s ability to arrest ferroptosis [77] and to augment the mitochondrial glutathione pool, enhance mitochondrial complex activity [53], mop up free radicals, and thereby reduce oxidative stress [78,79]. The antioxidant activity of levodopa observed in this study may be due to its dopamine synthesis-enhancing property [24,65].

## Conclusion

Ribose-cysteine and levodopa alone and in combination potentiated therapeutic effects on alpha-synuclein transgenic *Drosophila melanogaster* model of Parkinsonism. This is obvious in the ameliorative roles of these compounds against α-Synuclein-induced neurotoxicity and the derangement of several cellular physiological activities and behaviour.
